# Catalytically active tissue transglutaminase colocalises with Aβ pathology in Alzheimer’s disease mouse models

**DOI:** 10.1038/srep20569

**Published:** 2016-02-03

**Authors:** Micha M. M. Wilhelmus, Mieke de Jager, August B. Smit, Rolinka J. van der Loo, Benjamin Drukarch

**Affiliations:** 1 Department of Anatomy and Neurosciences, Neuroscience Campus Amsterdam, VU medical center, Amsterdam, The Netherlands; 2 Department of Molecular and Cellular Neurobiology, Center for Neurogenomics and Cognitive Research, VU University, Amsterdam, the Netherlands

## Abstract

Alzheimer’s disease (AD) is characterised by amyloid-beta (Aβ) protein deposition in the brain. Posttranslational modifications in Aβ play an important role in Aβ deposition. Tissue transglutaminase (tTG) is an enzyme involved in posttranslational cross-linking of proteins. tTG levels and activity are increased in AD brains, and tTG is associated with Aβ deposits and lesion-associated astrocytes in AD cases. Furthermore, Aβ is a substrate of tTG-catalysed cross-linking. To study the role of tTG in Aβ pathology, we compared tTG distribution and activity in both the APP_SWE_/PS1_ΔE9_ and APP23 mice models with human AD. Using immunohistochemistry, we found association of both tTG and *in situ* active tTG with Aβ plaques and vascular Aβ, in early and late stages of Aβ deposition. In addition, tTG staining colocalised with Aβ-associated reactive astrocytes. Thus, alike human AD cases, tTG was associated with Aβ depositions in these AD models. Although, distribution pattern and spatial overlay of both tTG and its activity with Aβ pathology was substantially different from human AD cases, our findings provide evidence for an early role of tTG in Aβ pathology. Yet, species differences should be taken into account when using these models to study the role of tTG in Aβ pathology.

Alzheimer’s disease (AD) is characterised by the aggregation of amyloid-β (Aβ) protein in brain parenchyma as senile plaques (SPs) and in blood vessel walls as cerebral amyloid angiopathy (CAA)^1^. In AD, Aβ shifts from soluble monomers to toxic oligomers and eventually forms insoluble mature fibrils^2^. Although it is known that posttranslational modifications in Aβ and Aβ chaperones, such as heparan sulphate proteoglycans, apolipoprotein E (ApoE), heat shock proteins, proteins of the complement system and transglutaminases that co-deposit with Aβ in SPs and CAA, influence Aβ aggregation^3^,^4^, their exact role in the underlying mechanisms leading to Aβ accumulation in the brain remains largely unknown.

The enzyme tissue transglutaminase (tTG) belongs to the family of calcium-dependent transglutaminases (TGs, EC 2.3.2.13), and has a role in signal transduction as a GTPase and in cell-matrix interactions by binding to integrins to facilitate cell adhesion and migration^5^. In addition, tTG also plays an important role in posttranslational modifications of proteins via amine incorporation and molecular cross-linking. The latter is formed by a γ-glutamyl-ε-lysine bond between a glutamine residue and a lysine residue of a peptide^6^. Although tTG is abundantly present in the brain, it is predominantly catalytically silent under physiological conditions^7^,^8^. In AD, the expression and activity of tTG is increased compared to controls^9^ and this correlates with cognitive decline in AD patients^10^,^11^. *In vitro* studies demonstrated that Aβ is a substrate for tTG-catalysed cross-linking, inducing Aβ oligomerisation and aggregation^12^,^13^,^14^,^15^,^16^. In previous work of our group, we demonstrated that tTG and its cross-links are not only present in both classic SPs and CAA in AD cases, but also colocalise with Aβ in diffuse SPs, suggested precursors of classic SPs, and early stages of CAA suggesting that tTG may be important in the onset of the Aβ cascade and/or early stages in the formation of SPs and CAA. In addition, tTG was present in reactive astrocytes associated with the above-described lesions^8^,^17^.

At present, knowledge concerning the expression, activity and distribution of tTG in AD is derived from post mortem human brain material and cerebrospinal fluid^9^,^10^,^18^,^19^,^20^,^21^. Although this provides valuable information on the possible role of tTG in Aβ pathology, investigating whether tTG might be a potential target to counteract Aβ pathology requires suitable animal models that mimic both the distribution and activity of tTG in development of Aβ pathology as observed in AD cases. For this purpose we selected two well-characterised AD mouse models, i.e. the APP_SWE_/PS1_ΔE9_ (APP/PS1) and APP23 mouse models, as they differ in the age of onset and duration of development of Aβ pathology. The APP/PS1 mice overexpress both the human APP Swedish mutation and the human presenilin 1 gene with deletion of exon 9 (PS1ΔE9)^22^. This leads to increased Aβ cleavage^23^ and Aβ plaques and some vascular amyloid are apparent from 4–6 months of age with high plaque burden from 12 months of age onwards^24^,^25^. Other AD characteristics, such as glial activation^26^ and memory deficits^27^, are found in these mice as well^26^,^28^,^29^. In contrast, the APP23 mice demonstrate a later onset and slower progression of Aβ pathology. APP23 mice overexpress the human APP Swedish mutation and are characterised by initial rare Aβ plaques at 6 months of age and vascular amyloid from 12 months of age^30^,^31^. At 24 to 27 months of age, mice show extensive Aβ pathology, both Aβ plaques and vascular amyloid, covering a substantial area of the cortex^31^,^32^. Also other AD hallmarks, such as neuronal loss, glial activation^31^ and early cognitive deficits^33^,^34^ are described. Thus, both models provide insight in the development of Aβ pathology, albeit with differences in form, onset and development of Aβ pathology and its severity. As we were interested in the role of tTG in AD pathology and in particular Aβ aggregation and accumulation, in the present study we investigated whether the distribution of tTG and *in situ* activity in these AD mouse models at different stages of Aβ pathology is similar to our observations in human AD cases. For this purpose, we used immunohistochemistry and an *in situ* tTG activity assay on post-mortem tissue sections.

## Results

### Distribution of Aβ pathology in transgenic mice

**Figure 1:**
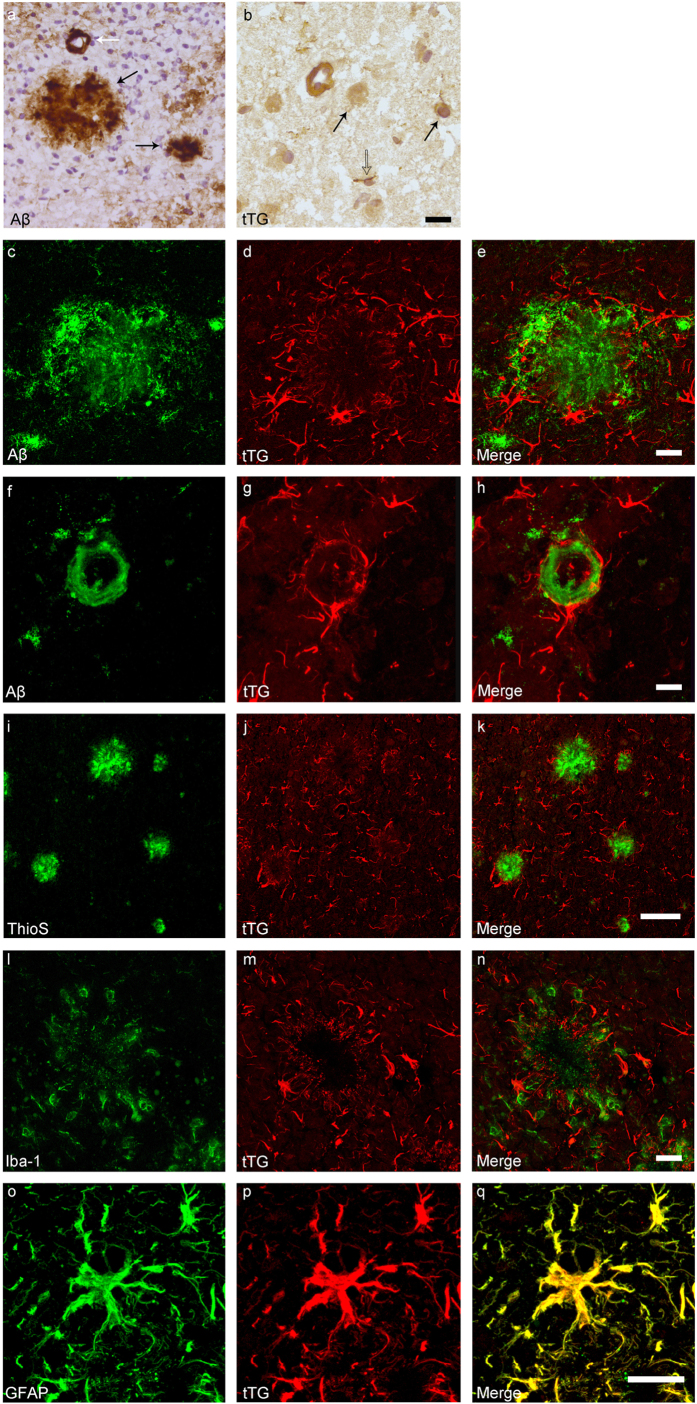
Distribution of the anti-Aβ antibody and anti-tTG antibodies in sagittal whole brain sections of C57Bl6/J wild-type and APP23 mice brains. 27-months old APP23 mice demonstrated Aβ plaques (**a**, black arrow) and vascular Aβ (**a**, white arrow). Anti-tTG antibody immunoreactivity was observed in blood vessel walls (**b**) as well as neurons (**b**, black arrow) and in glial cells (**b**, black open arrow) in both 27-months old wild-type (**b**) and 27-months old APP23 mice (not shown). In APP23 mice, additional tTG antibody immunoreactivity was present in glial cells associated with Aβ plaques (**c–e**) and vascular Aβ (**f–h**) in 24/27-months old mice. Anti-tTG antibody immunoreactivity was associated with the majority of ThioS positive plaques (**i–k**). Double immunofluorescence of the anti-Iba-1 antibody or the anti-GFAP antibody with the anti-tTG antibody demonstrated absence of tTG in Iba-1 positive microglia (**l–n**), whereas tTG immunoreactivity colocalised with GFAP positive astrocytes (**o–q**). Scale bars: (**a**) 20 μm, (**b**,**f**–**h**,**l**–**q**) 15 μm, (**c**–**e**) 30 μm, (**i**–**k**) 60 μm. Abbreviations: Aβ = amyloid beta, GFAP = glial fibrillary acidic protein, Iba-1 = Ionized calcium binding adaptor molecule 1, ThioS = Thioflavin S, tTG = tissue transglutaminase.

In the neocortex of 12-months old APP23 mice, the anti-Aβ antibody demonstrated presence of few Aβ plaques and Aβ-affected vessels, whereas in older (24- or 27- months old) mice the number of Aβ deposits in both plaques (Fig. 1a, black arrows) and vessels (Fig. 1a, white arrow) had increased. In addition to Aβ pathology as observed in the cortex of young mice, older mice also demonstrated Aβ pathology in the hippocampus and thalamus, which is in line with previous research^30^. In APP/PS1 mice, we found Aβ deposits in neocortex and hippocampus of 7-months old mice with an increase in number of Aβ plaques in 12-months old mice, whereas only few vessels displayed vascular Aβ deposits (not shown), which is in line with earlier findings^24^,^25^,^29^. In addition, in these older APP/PS1 mice a few Aβ plaques and vascular Aβ were present in the cerebellum and thalamus as well (not shown). In both mouse models, double immunofluorescence of ThioS with the anti-Aβ antibody confirmed the presence of both dense-core β-pleated sheet plaques (ThioS positive) and diffuse plaques (anti-Aβ antibody positive and ThioS negative), although the Aβ plaques in 12-months old APP23 mice were predominantly ThioS positive Aβ depositions^35^,^36^. In addition, as described previously^26^,^31^, glial cells were present surrounding the Aβ plaques and CAA in both mouse models (not shown).

### Association of tTG with Aβ pathology in transgenic mice

**Figure 2:**
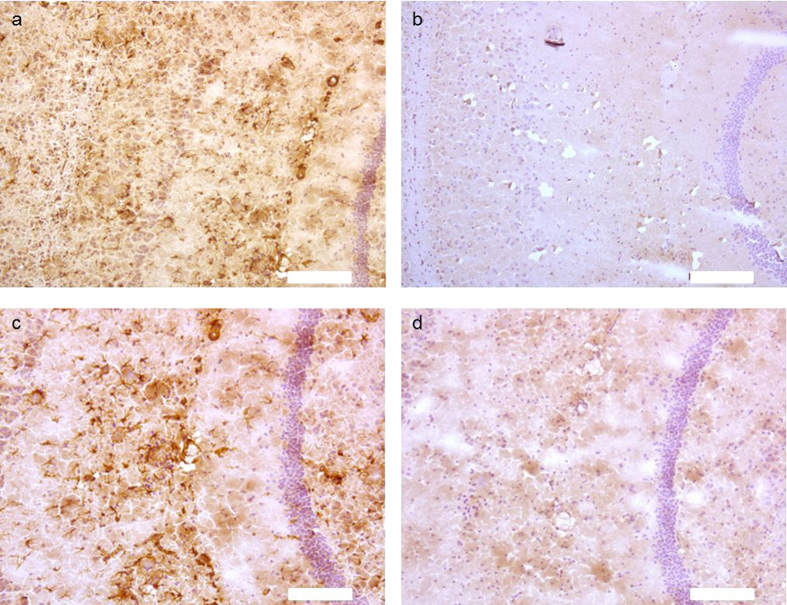
Anti-tTG antibody specificity in sagittal whole brain sections of APP23 mice brains. High power magnification of anti-tTG antibody staining in 24-months old APP23 mice brain sections showing distribution in neurons, cerebral vessels, glial cell and Aβ deposits (**a,c**). Omission of the primary anti-tTG antibody in 24-months old APP23 mice brain using serial sections (**a,b**) demonstrated the absence of staining (**b**). Preadsorption of the anti-tTG antibody with its antigen Guinea pig tTG demonstrated the absence of staining in neurons, cerebral vessels, glial cell and Aβ deposits in serial sections (**c,d**). Scale bars (**a–d**): 200 μm.

In previous work of our group, we demonstrated that in human brain of control and AD cases the anti-tTG antibody (06471) stained the cytoplasm of neuronal cells and colocalised with cerebral vessels and glial cells, and the pathological hallmarks of AD, i.e. cerebral amyloid angiopathy and senile plaques^17^,^37^. The anti-tTG antibody (06471) also demonstrated a similar distribution pattern in human brain as the monoclonal anti-tTG antibody Ab-1 (LabVision)^17^,^37^. To demonstrate anti-tTG antibody specificity in mice brain, we first analysed aspecific staining of the secondary antibody. Omission of the primary anti-tTG antibody demonstrated the absence of staining in 24-month old APP23 mice (Fig. 2a,b). Preadsorption of the anti-tTG antibody with its antigen Guinea pig tTG demonstrated the absence of staining in neurons (Figs 1b and 2c,d, black arrow), throughout the blood vessel walls of all cerebral vessels, i.e. leptomeningeal and parenchymal vessels and capillaries (Figs 1b and 2c,d), glial cells (Figs1o–q and 2c,d,) and all Aβ deposits (Figs1c–e and 2c,d), which is in line with observations in human post-mortem brain tissue^7^,^8^. No differences in general tTG distribution or staining intensity were observed between age groups. In addition, tTG staining was also observed in glial cells associated with vascular Aβ (Fig. 1f–h), although no difference in localisation or staining intensity with non-Aβ laden vessels was observed. In APP/PS1 mice, both 7- and 12-months old, only little vascular Aβ was present, hampering extensive comparison for tTG staining (not shown). Double immunofluorescence of the anti-tTG antibody with ThioS showed association of anti-tTG antibody immunoreactivity with all ThioS positive plaques in both APP23 (Fig. 1i–k) and APP/PS1 mice. In APP/PS1 mice only, tTG staining was also present in Aβ plaques negative for ThioS and positive for anti-Aβ antibody immunoreactivity (not shown). In general, we found a similar distribution pattern of anti-tTG antibody immunoreactivity between different age groups. However, a higher number of tTG-positive astrocyte-like cells were found in both 12-months old APP/PS1 and 24/27-months old APP23 mice, likely due to increased Aβ plaque load. Identification of the cellular source of tTG in Aβ plaque-associated cells was performed using double immunofluorescence of the astrocyte marker glial fibrillary acidic protein (GFAP), or the microglial marker ionized calcium binding adaptor molecule 1 (Iba-1) with tTG. In contrast to microglial cells (Fig. 1l–n), we found extensive colocalisation of tTG staining with GFAP-positive reactive astrocytes (Fig. 1o–q) associated with Aβ deposits in both mouse models.

### Association of *in situ* active TG with Aβ pathology in transgenic mice

**Figure 3:**
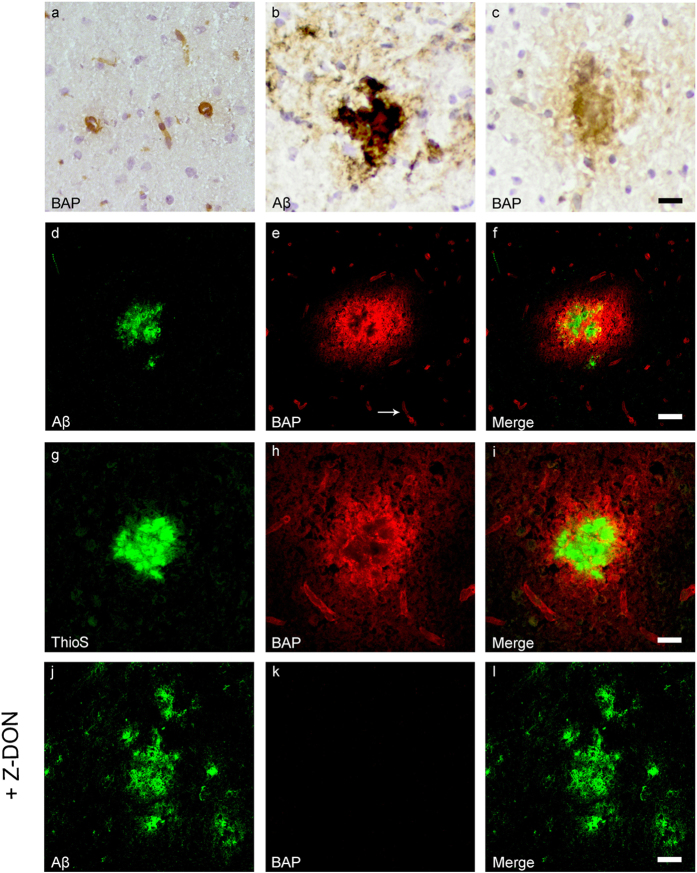
Distribution of *in situ* TG activity in C57Bl6/J wild-type and APP23 mice. Serial sagittal whole brain sections of wild-type and APP23 mice were incubated with the TG substrate BAP or the anti-Aβ antibody and visualised using the DAB chromogen. The anti-Aβ antibody demonstrated Aβ plaques in 27-months old mice (**b**). BAP staining was found in the cerebral blood vessel walls of both 7-months old wild-type (**a**) and 27-months old APP23 mice (**c**). In addition, in APP23 mice BAP staining was present in Aβ plaque-like structures (**c**). Double immunofluorescence using the anti-Aβ antibody and BAP staining confirmed the presence of TG activity in Aβ plaques in 12-months old mice (**d–f**) as well as in cerebral vessel walls (**e**, arrow). BAP staining was found in the majority of dense core plaques, although it was absent from the cores of these plaques, confirmed by double immunofluorescence of ThioS with BAP (**g–i**). Co-incubation of BAP with the selective tTG inhibitor Z-DON (100 μM) blocked the tTG-catalysed incorporation of BAP (**j–l**). Scale bars: (**c**,**d**) 36 μm, (**a**,**d**–**f**,**j**–l) 30 μm, (**g**–**i**) 15 μm. Abbreviations: Aβ = amyloid beta, BAP = biotinylated 5-(biotinamido)-pentylamine, ThioS = Thioflavin S, tTG = tissue transglutaminase, Z-DON = Z-DON-Val-Pro-Leu-OMe.

**Figure 4:**
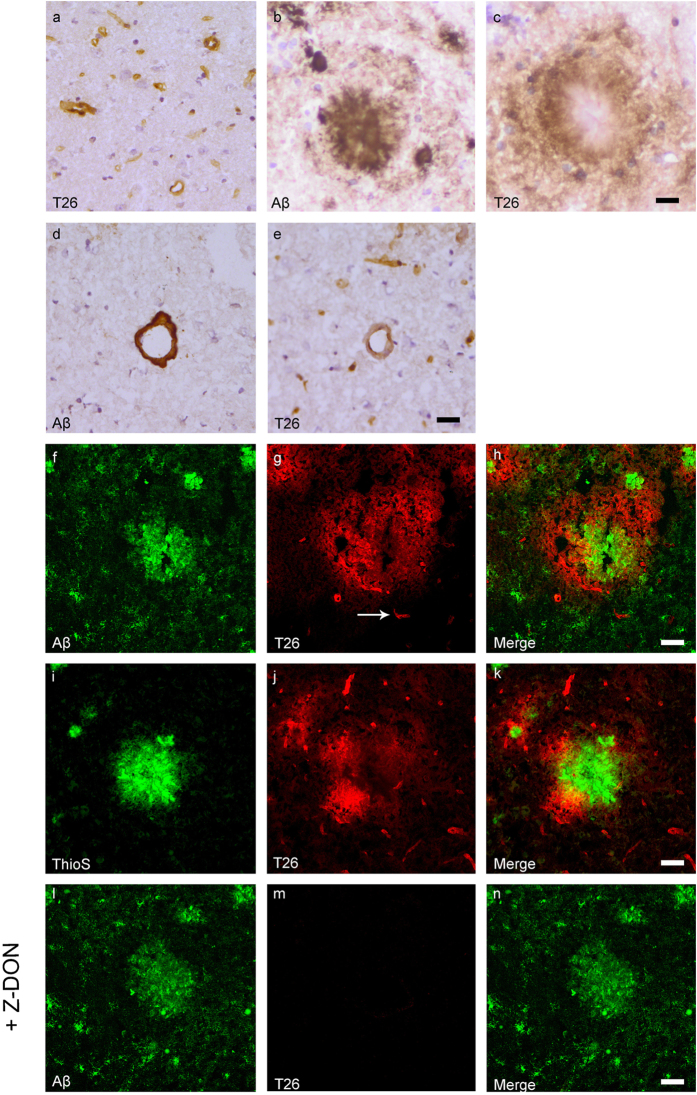
Distribution of *in situ* tTG activity in sagittal whole brain sections in C57Bl6/J wild-type and APP23 mice brains. Sagittal serial brain sections of mice were incubated with the specific tTG substrate T26 or the anti-Aβ antibody and visualised using the DAB chromogen. The anti-Aβ antibody stained both plaques (**b**) and vascular Aβ (**d**) in 27-months old mice. T26 staining was present in cerebral blood vessel walls in both 27-months old wild-type (**a**) and APP23 mice (**g**, arrow). In addition, in 27-months old APP23 mice, T26 stained both Aβ plaques (**c**) and in vascular Aβ (**e**). Double immunofluorescence of the anti-Aβ antibody with T26 staining demonstrated colocalisation of T26 with Aβ plaques (**f–h**). T26 staining colocalised with the majority of ThioS positive plaques, although T26 staining was absent from the dense cores of these plaques (**i–k**). Co-incubation of T26 with the selective tTG inhibitor Z-DON prevented the tTG-catalysed incorporation of T26 (**l–n**). Scale bars: (**a**–**c**) 20 μm, (**d**–**n**) 30 μm. Abbreviations: Aβ = amyloid beta, ThioS = Thioflavin S, tTG = tissue transglutaminase, Z-DON = Z-DON-Val-Pro-Leu-OMe.

*In situ* endogenous TG activity was analysed by measuring the incorporation of both the TG substrates BAP and T26^38^. BAP and T26 are a primary amine and a glutamine-bearing peptide, respectively, and are thus involved in different steps of the tTG-catalysed cross-link reaction. Therefore, we used both BAP and T26 as a control for the specificity of the *in situ* tTG activity staining. In general, in both wild-type (Figs 3a and 4a) and transgenic mice (Figs 3c and 4c) we observed presence of both BAP (Fig. 3) and T26 (Fig. 4) throughout the vessel walls of both leptomeningeal and parenchymal vessels, as well as in capillaries. No difference was observed between different age group in BAP or T26 distribution and/or staining intensity. In addition, in both the transgenic lines, BAP and T26 staining was also associated with a subset of anti-Aβ antibody positive plaques as demonstrated using serial sections (Figs 3b,c and 4b,c, respectively) and double immunofluorescence (Figs 3d–f and 4f–h, respectively). In vascular Aβ deposits, both BAP (not shown) and T26 (Fig. 4d,e) staining was present, although no difference in staining was found compared to control vessels in both transgenic mouse models. In addition, double immunofluorescence of ThioS with BAP or T26 demonstrated association of *in situ* active tTG with vascular Aβ (not shown) and a subset of Aβ plaques (Figs 3g–i and 4i–k, respectively), although in the dense cores of these plaques, no *in situ* active tTG was found. In addition, in APP/PS1 mice, we also found BAP and T26 staining in ThioS negative, but anti-Aβ antibody positive, Aβ plaques (not shown). In 12-months old APP23 mice, however, only a few anti-Aβ antibody or ThioS positive plaques were present of which only a subset demonstrated BAP or T26 incorporation (not shown).

To determine tTG-specific incorporation of both BAP and T26 into proteins present in the post-mortem tissue, we co-incubated BAP or T26 with the selective irreversible tTG inhibitor Z-DON^34^,^35^. Co-incubation of Z-DON with BAP or T26 inhibits the incorporation of both substrates and resulted in absence of staining (Figs 3j–l and 4l–n respectively).

### Semi-quantification of percentage of BAP and T26 positive plaques

**Figure 5:**
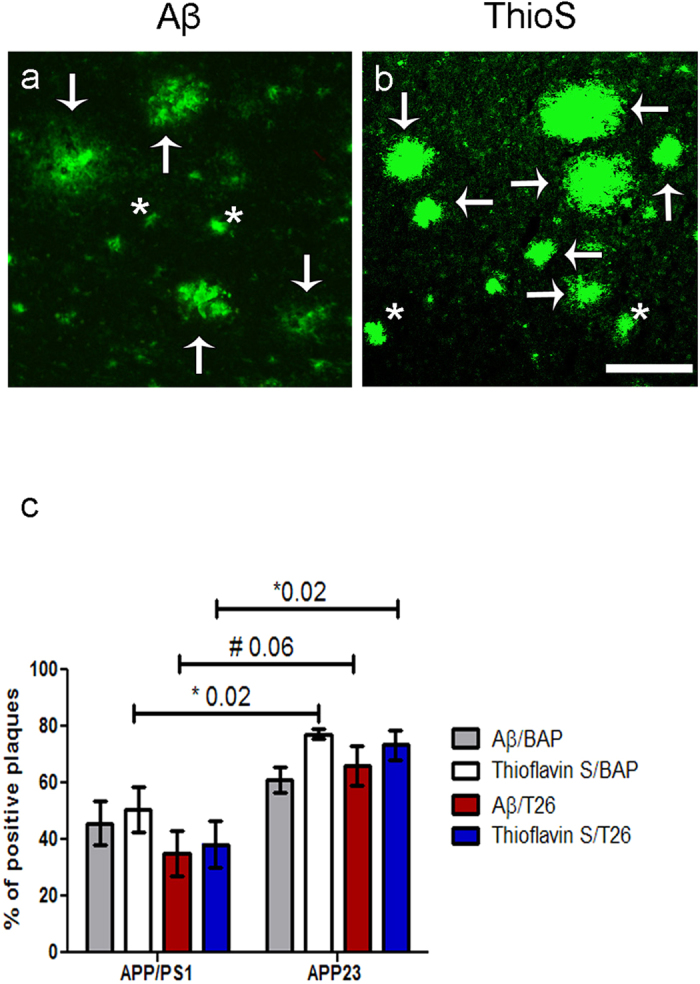
Quantification of the percentage of anti-Aβ antibody positive and ThioS positive plaques with BAP or T26 staining was performed in 12-months old APP/PS1 mice and 24- and 27-months old APP23 mice. Double immunofluorescence was performed with an anti-Aβ antibody or ThioS with BAP or T26 resulting in double stainings Aβ/BAP, Aβ/T26, ThioS/BAP and ThioS/T26. Only well-defined anti-Aβ antibody positive or ThioS-positive plaques were counted. Well-defined plaques are marked with arrows, both for Aβ (**a**) and ThioS (**b**) staining (APP23 staining shown), whereas examples of plaques that were not taken into quantification are marked with asterisks (**a,b**). The percentages of anti-Aβ antibody positive and ThioS positive plaques positive for BAP or T26 are shown for both APP23 and APP/PS1 mice (**c**). Non-parametric Kruskal-Wallis testing demonstrated a significant higher percentage of ThioS positive plaques with BAP (77.3 ± 1.9% Mean ± SEM) or T26 (73.4 ± 5.2%) staining in APP23 mice compared to APP/PS1 mice where BAP and T26 staining were present in 50.5 ± 8.0% or 38.3 ± 8.1% of the ThioS positive plaques respectively (p = 0.02). In APP23, a trend increase of the percentage of Aβ plaques with T26 staining was present compared to APP/PS1 mice (66.1 ± 6.9% versus 35.5 ± 8.0% respectively, p = 0.06). The increased percentage of Aβ plaques with BAP staining in APP23 mice (61.0 ± 6.9%) compared to APP/PS1 mice (45.7 ± 7.8%) did not reach statistical significance (Fig. 4c). Error bar: 300 μm. Abbreviations: Aβ = amyloid beta, BAP = biotinylated 5-(biotinamido)-pentylamine ThioS = Thioflavin S, tTG = tissue transglutaminase. *p < 0.05, ^#^p < 0.1. Mean ± SEM is displayed.

In order to gain more insight into the differences in *in situ* active tTG staining between both mouse models, we quantified the percentage of anti-Aβ antibody positive plaques (Fig. 5a) and ThioS positive plaques (Fig. 5b) that demonstrated BAP or T26 staining (Fig. 5c). Non-parametric Kruskal-Wallis testing demonstrated a significant higher percentage of ThioS positive plaques with BAP (77.3 ± 1.9% Mean ± SEM) or T26 (73.4 ± 5.2%) staining in APP23 mice compared to APP/PS1 mice where BAP and T26 staining were present in 50.5 ± 8.0% or 38.3 ± 8.1% of the ThioS positive plaques, respectively (*p* = 0.02). In APP23, an increase in the percentage of Aβ plaques with T26 staining was present compared to APP/PS1 mice (66.1 ± 6.9% versus 35.5 ± 8.0% respectively, *p* = 0.06). In addition, although we found an increased percentage of Aβ plaques with BAP staining in APP23 mice (61.0 ± 6.9%) compared to APP/PS1 mice (45.7 ± 7.8%), the difference did not reach statistical significance. In addition, one-way ANOVA test showed no statistical differences in tTG activity between the different stainings within each mouse model.

## Discussion

In this study, we describe for the first time the association of tTG with Aβ pathology in two different AD mouse models. Interestingly, although the general distribution pattern of tTG in mice was similar to our observations in human brain, the association of tTG and *in situ* active tTG with Aβ pathology differed between the mouse models and human AD cases. In both mouse models, the anti-tTG antibody and the *in situ* tTG activity staining was associated with Aβ staining but did not overlay spatially with the deposited Aβ, which is in contrast to our observations in human AD cases^7^,^17^,^39^. Furthermore, between both mouse models differences were also observed, as a comparison between the association of *in situ* active tTG with Aβ pathology demonstrated that the majority of ThioS positive plaques in APP23 mice showed tTG activity, whereas less activity was present in APP/PS1 mice plaques. However, in line with our observation in human AD cases, association of tTG with Aβ pathology was already observed in early forms of Aβ pathology formation i.e. diffuse plaques. Thus, alike the findings in human AD, our data in AD mouse models hint towards a role for tTG in Aβ pathology, although the differences in tTG distribution and *in situ* activity between these models and human AD cases indicate that these AD models only partly mimic tTG’s role in Aβ pathology in human AD cases.

AD mouse models are limited in their translation to human disease, especially sporadic AD. All current AD mouse models are generated with mutations in the human *APP* and *PSEN* genes that drive Aβ production and deposition, whereas only 1% of AD cases are linked to mutations. In addition, although AD mouse models show Aβ deposition, the chemical properties of these depositions differ significantly. This is for instance illustrated by the fact that posttranslational modifications of Aβ, isomerisation and truncations, as well as the colocalisation of Aβ chaperones with Aβ deposits in AD, are either lacking in mouse models or differ from human AD^40^,^41^,^42^,^43^. In this study, we indeed found some differences between the presence and *in situ* activity of tTG in the studied mouse models compared to human AD. Using immunohistochemistry with anti-tTG antibodies, tTG colocalised with the Aβ deposition in SPs in human AD, whereas in both mouse models, tTG did not colocalise with deposited Aβ. This may be a consequence of the rapid development of the Aβ pathology in mice compared to human AD, which may not allow enough time for tTG to co-deposit in the Aβ plaque. Furthermore, although in human SPs we observed no *in situ* tTG activity (unpublished observations), in both mouse models *in situ* tTG activity was present surrounding the cores of Aβ plaques. These data indicate the presence of tTG in deposited Aβ plaques in the mouse models, but the levels or conformation of tTG may be too low or unsuitable for anti-tTG antibody detection. In the vascular depositions of Aβ, discrepancies between distribution of tTG in human AD cases and AD mouse models were also observed. In CAA in human AD, tTG did not colocalise with the Aβ deposition per se, but was present in two halos surrounding the Aβ deposition and *in situ* TG activity was increased in CAA^17^. In both AD mice models, however, tTG immunoreactivity and *in situ* activity was present in the vessel walls of all brain vessels regardless of Aβ pathology. These data suggest that CAA as found in human AD cases is not or only partly mimicked by the vascular Aβ deposits observed in mouse models. Indeed several posttranslational modifications of Aβ, such as pyroglutamate-modified Aβ, that affect Aβ aggregation, stability and toxicity^44^,^45^,^46^, are present in CAA of human AD^46^ but absent from vascular Aβ depositions in APP23 mice^40^. The different composition may affect tTG distribution and activity although the exact mechanisms require further research. In conclusion, association of tTG and its activity with Aβ pathology in both mouse models in both early and late stages of Aβ pathology formation is largely different from human AD, suggesting that these mouse models do not fully represent the role of tTG in human AD. However, alike human AD^8^,^17^, in the present study, we found colocalisation of tTG with Aβ deposition-associated astrocytes in both mouse models. This suggests that tTG has a similar role in astrocytes associated with Aβ pathology in these mouse models as in human AD.

Pathological conditions such as tissue damage and inflammatory conditions lead to an increase in tTG expression^47^. In AD, Aβ deposition results in an inflammatory response by activation of glial cells^48^. Previously we observed tTG and its cross-links in Aβ-associated reactive astrocytes^17^,^39^ suggesting that tTG is upregulated by the activation of these cells^11^. Here, a similar phenomenon was observed, as a strong increase of tTG staining in astrocytes associated with Aβ plaques and vascular Aβ deposition in both mouse models was demonstrated. At sites of Aβ depositions reactive astrocytes are attracted to the injury site to form a protective barrier that prevents the surrounding tissue, including neurons, from damage and inflammation^48^. Importantly, it was previously shown *in vitro* that tTG is involved in the migration of astrocytes^49^. Thus, Aβ deposition may trigger the recruitment of astrocytes via upregulation of tTG. Subsequently, astrocytes may secrete tTG into the extracellular space where it can become active and play a role in the promotion of tissue repair^50^. However, although in both mouse models and AD cases a similar presence and activity of tTG in astrocytes associated with Aβ depositions has thus been demonstrated by us, more research is necessary to demonstrate whether the tTG in reactive astrocytes in human AD and in these mouse models is directly triggered by Aβ or via tissue damage evoked by Aβ deposition.

Aβ deposition in human brain parenchyma is suggested to start with deposition as diffuse plaques that over time progress into dense-core ‘classic’ plaques^51^. CAA development starts with Aβ deposition in the medial layer of the vessel walls which progresses into all layers of the vessel wall^52^. Previously we demonstrated that tTG staining is present in these early forms of Aβ deposition, as we observed the presence of tTG in diffuse plaques^8^ and in early-stage CAA^17^. Similar to our observations in human AD cases, in the present study we observed the presence of tTG in astrocytes associated with diffuse plaques in APP/PS1 mice. Although not abundantly, astrocytes are present surrounding diffuse plaques in human AD^48^, thus indicating that astrocyte-derived tTG may be upregulated in early Aβ depositions. In addition to the presence of tTG in diffuse Aβ pathology, we demonstrated that at ages at which Aβ pathology starts to form (7- and 12-months old APP/PS1 and APP23 mice, respectively^24^,^30^, tTG is already associated with the Aβ depositions. In APP23 mice, however, tTG staining was absent from diffuse plaques. A possible explanation for this discrepancy is that we and others^35^,^36^ only observed diffuse plaques in older APP23 mice, suggesting that in these mice diffuse plaques do not represent an early form of Aβ deposition or are indicative of an alternative Aβ aggregation pathway. Together, these data indicate that tTG might play a role at the onset of Aβ deposition.

Although both mouse models demonstrated association of *in situ* tTG activity with Aβ depositions, a clear difference in the levels of association between both models was observed. The percentage of plaques associated with tTG activity was significantly higher in APP23 mice compared to APP/PS1 mice. An important difference between both mouse models is neuronal cell death. In the APP/PS1 mouse model, neuronal cell death is not observed^53^, whereas it is present in the hippocampus of APP23 mice^35^. Interestingly, elevated tTG levels and activity are associated with increased cell death^60^. tTG may promote apoptosis pathways and is involved in stabilising dying cells by cross-linking of intracellular components to inhibit leakage and prevent scarring and inflammation^54^. In addition, cell death is accompanied by an inflammatory response^55^ that may also increase tTG levels and activity^47^. Thus, the observed increase in overall tTG activity associated with Aβ pathology in APP23 compared to the APP/PS1 mice might be caused directly and/or indirectly by neuronal cell death found in these mice.

In conclusion, although there is one publication that argues against a role for tTG in AD^56^, mounting evidence provided by us and others increasingly argues in favour of a important role for tTG in Aβ pathology^7^,^8^,^12^,^17^,^21^,^39^,^57^,^58^,^59^,^60^,^61^. Here, we demonstrated the association of both tTG protein as well as enzyme activity with Aβ pathology in two well-known AD mouse models. In addition, we confirm our immunohistochemical findings in human post-mortem AD tissue that tTG is already associated with these Aβ lesions in early stages of their development. However, the exact distribution of both tTG enzyme and its *in situ* activity differs substantially between AD mouse models and human AD cases. Therefore, the above-described species differences should be taken into account when using these models to unravel the exact role of tTG in Aβ pathology.

## Materials and Methods

### Mice

APP_SWE_/PS1_ΔE9_ (APP/PS1) mice were generated on a C57Bl/6 background as described previously^23^. In short, mice overexpress the human APP_695_ splice variant containing the Swedish mutation at position 595/596 as well as the ΔE9 mutation in the human presenilin 1 (PS1) gene under the mouse prion promoter^23^. APP23 transgenic mice were also generated on a C57Bl/6 background, as described previously^31^. APP23 mice overexpress the human APP_751_ splice variant containing the Swedish double mutation at positions 670/671 under the murine modified Thy1.2 promoter^31^. All used control animals were C57Bl/6.

### Brain tissue

Brains of six 7-months old heterozygous APP/PS1 mice and six age-matched controls (all male), as well as six 12-months old APP/PS1 mice (4 male, 2 female) and five age-matched controls (all male) were used. APP/PS1 mice were sacrificed using cervical dislocation and brains were directly isolated and snap-frozen in liquid nitrogen. Approval was obtained from the animal ethics committee of the VU University.

In addition, brain hemispheres of four 12-months old heterozygous APP23 mice (2 male, 2 female) and one age-matched control mouse (male) as well as four 24- or 27-months old heterozygous APP23 mice and one age-matched control (all male) were kindly provided by Prof. Dr. Matthias Jucker, Department of Cellular Neurology, Hertie Institute for Clinical Brain Research and German Center for Neurodegenerative Diseases, University of Tübingen, Tübingen, Germany and Dr. Matthias Staufenbiel, Novartis Institutes for Biomedical Research, Basel, Switzerland. Mice were transcardially perfused with PBS, brains were isolated and snap-frozen in liquid nitrogen. The experimental procedures using mice were carried out in accordance with the veterinary office regulations of Baden-Württemberg (Germany) and approved by the local Animal Care and Use Committees. All experiments were performed in accordance with relevant guidelines and regulations.

### Immunohistochemistry

Immunohistochemistry for mouse brain tissue was performed as described previously by us for human brain tissue^8^,^17^. Sagittal sections (6 μm) of all above-described animals were fixed with acetone (100%) for 10 minutes, or 4% paraformaldehyde for 20 minutes (only used for Iba-1 staining, described below). Endogenous peroxidase activity was blocked with 0.3% hydrogen peroxide, 0.1% sodium azide in Tris buffered saline (TBS) pH 7.6 for 15 minutes. Subsequently, sections were blocked with 3% bovine serum albumin (BSA; PAA Laboratories, Pasching, Austria) in TBS with 0.5% TritonX-100 (TBS-T). Primary antibodies (Table 1) were diluted in 3% BSA/TBS-T and incubated overnight at 4 °C. Negative controls were incubated in this solution without the primary antibodies. The secondary antibodies, biotinylated goat anti-rabbit or donkey anti-goat (dilution 1:400; Jackson Immunoresearch Laboratories Inc., Suffolk, UK) were diluted in 3% BSA/TBS-T and incubated for 2 hours at room temperature followed by incubation with the avidin-biotin complex (ABC, 1:400 in TBS/T; Vector Laboratories Inc., Burlingame, CA, USA) for 1 hour. Between incubation steps, sections were extensively washed with TBS. Stainings were visualised with 0.05% 3,3′-diaminobenzidine (DAB) with 0.01% hydrogen peroxide in Tris-hydrochloride (Tris-HCl buffer, pH 7.6). Sections were rinsed with Tris-HCl and tap water, counterstained with haematoxylin nuclear dye and washed with tap water. Sections were dehydrated using a series of increasing alcohol dilutions followed by xylene. Sections were coverslipped with Entellan® mounting medium (Merck Millipore, Darmstadt, Germany) and examined with an Olympus Vanox light microscope (Olympus Microscopy, Hamburg, Germany) or a Leica CTR5000 light microscope (Leica Microsystems, Rijswijk, the Netherlands). Staining was performed and analyzed on at least three sections per animal. In the figures, a representative image of these stainings is shown. The specificity of the 06471 antibody was demonstrated by preadsortpion by its antigen, i.e. purified liver Guinea pig tTG (Sigma-Aldrich, Zwijndrecht, The Netherlands). The negative control for the secondary donkey anti-goat antibody consisted of representative sections processed without the primary antibody.

### Double immunofluorescence

Sections of all above-described animals were fixed and stained as described above, excluding the endogenous peroxidase blocking step, ABC step and DAB visualisation. Primary antibodies are listed in Table 1. Secondary antibodies used were donkey anti-goat and donkey anti-rabbit, both coupled to either Alexa 488 or Alexa 594, (dilution 1:400, Invitrogen, Camarillo, CA, USA). For the detection of β-pleated sheets, sections were incubated with 1% Thioflavin S (ThioS, Sigma, St. Louis, Missouri USA) in milliQ for 5 minutes, washed three times with 70% ethanol and two times with TBS. Sections were mounted with Vectashield® (Vector laboratories Inc) or PVA-DABCO^®^ mounting medium (Sigma). Between incubation steps, sections were washed extensively with TBS. A Leica TCS SP2 AOBS confocal laser scanning microscope (Leica Microsystems) was used to visualise the immunofluorescence. To exclude bleed-through of fluorescence emission, a series of images was obtained by sequential scanning of channels through a 40× lens (zoom factor 1× or 2×, resolution 1024 × 1024). Staining was performed and analyzed on at least three sections per animal. In the figures, a representative image of these stainings is shown.

### *In situ* TG activity

*In situ* TG activity detection was performed as described previously^17^. In short, unfixed 6μm thick tissue sections of APP23, APP/PS1 mice and their age-matched controls were pre-incubated for 20 minutes at room temperature in a 100 mM Tris-HCl, pH 7.4, 5 mM CaCl_2_, 1 mM dithiothreitol (DTT, Promega, Leiden, The Netherlands) buffer with or without 100 μM of the tTG activity inhibitor Z-DON-Val-Pro-Leu-OMe (Z-DON)^34^,^35^, purchased from Zedira GmbH, Darmstadt, Germany. Then, incubation was continued for 30 minutes at 37 °C with the same incubation buffer with or without inhibitor to which 50 μM of the general TG substrate biotinylated 5-(biotinamido)-pentylamine (BAP; Thermo Fisher Scientific, Waltham, MA, USA) or 50 μM of the specific tTG substrate T26 (Covalab, Villeurbanne, France) was added^38^,^62^. Thereafter, sections were air dried, fixed for 10 minutes with 100% acetone, blocked with 3% BSA/TBS-T and subsequently incubated with a primary antibody directed against Aβ in 3% BSA/TBS-T at 4 °C overnight followed by 2 hour incubation at room temperature with secondary antibodies donkey anti-rabbit coupled to Alexa 488 to detect Aβ (dilution 1:400) and streptavidin coupled to Alexa 594 to detect BAP or T26 incorporation (dilution 1:400). In case of double staining with ThioS, only incubation with streptavidin Alexa 594 was performed after blocking with 3% BSA/TBS-T followed by 1% ThioS as described above. Sections were washed with TBS in between and after antibody incubation and mounted with Vectashield or PVA-DABCO^®^ mounting medium (Sigma). The Leica TCS SP2 AOBS confocal laser-scanning microscope was used to visualise the staining, as described above. The tTG activity staining was performed and analyzed in at least three sections per animal. In the figures, a representative image of these stainings is shown.

### Semi-quantification of BAP or T26 positive Aβ plaques

In order to quantify the percentage of Aβ plaques positive for BAP or T26 staining, we performed double immunofluorescence of either ThioS or the anti-Aβ antibody with either BAP or T26. Pictures (zoom 10X) were taken throughout the brain sections of the mice. Only well-defined Aβ/ThioS plaques were counted and the percentage of plaques with BAP or T26 staining was calculated. Well-defined plaques were defined as plaques with a clear demarcated, plaque-like shape. We only quantified the 12-months old APP/PS1 and the 24/27-months old APP23 mice, as younger mice of both models only demonstrated few plaques with high inter-animal variation. A one-way ANOVA was performed to test group differences within a mouse model. For this, percentage values for the APP23 mice were log-transformed to obtain normally distributed values. Differences between the two mouse models were tested with a non-parametric Kruskal-Wallis test, without transformation of the data.

### Statistics

Data are displayed with Graphpad Prism 5 statistical software package and analysed with SPSS Statistics 20.0. P-values of ≤0.05 were regarded as statistically significant.

## Additional Information

**How to cite this article**: Wilhelmus, M. M. M. *et al.* Catalytically active tissue transglutaminase colocalises with Aβ pathology in Alzheimer's disease mouse models. *Sci. Rep.* **6**, 20569; doi: 10.1038/srep20569 (2016).

## Figures and Tables

**Table 1:** Primary antibodies.

Antigen	Primary antibody	Species raised in	Dilution	Fixation	Company
Aβ	Human Aβ (715800)	Rabbit	1:100	Acetone	Invitrogen, Camarillo, CA, USA
tTG	Guinea pig tTG (06471)	Goat	1:4000	Acetone	Millipore, Temecula, CA, USA
Astrocytes	Bovine Glial Fibrillary Acidic Protein	Rabbit	1:4000	Acetone	DAKO, Glostrup, Denmark
Microglia	Iba-1 (019–19741)	Rabbit	1:500	PFA	WAKO Chemicals, Richmond, VA, USA

Abbreviations: Aβ = amyloid beta, Iba-1 = Ionized calcium binding adaptor molecule 1, PFA = paraformaldehyde, tTG = tissue transglutaminase.
